# International Society of Sports Nutrition position stand: creatine supplementation and exercise

**DOI:** 10.1186/1550-2783-4-6

**Published:** 2007-08-30

**Authors:** Thomas W Buford, Richard B Kreider, Jeffrey R Stout, Mike Greenwood, Bill Campbell, Marie Spano, Tim Ziegenfuss, Hector Lopez, Jamie Landis, Jose Antonio

**Affiliations:** 1 International Society of Sports Nutrition, 600 Pembrook Drive, Woodland Park, CO 80863, USA

## A Position Statement and Review of the Literature

Position Statement: The following nine points related to the use of creatine as a nutritional supplement constitute the Position Statement of the Society. They have been approved by the Research Committee of the Society.

1. Creatine monohydrate is the most effective ergogenic nutritional supplement currently available to athletes in terms of increasing high-intensity exercise capacity and lean body mass during training.

2. Creatine monohydrate supplementation is not only safe, but possibly beneficial in regard to preventing injury and/or management of select medical conditions when taken within recommended guidelines.

3. There is no scientific evidence that the short- or long-term use of creatine monohydrate has any detrimental effects on otherwise healthy individuals.

4. If proper precautions and supervision are provided, supplementation in young athletes is acceptable and may provide a nutritional alternative to potentially dangerous anabolic drugs.

5. At present, creatine monohydrate is the most extensively studied and clinically effective form of creatine for use in nutritional supplements in terms of muscle uptake and ability to increase high-intensity exercise capacity.

6. The addition of carbohydrate or carbohydrate and protein to a creatine supplement appears to increase muscular retention of creatine, although the effect on performance measures may not be greater than using creatine monohydrate alone.

7. The quickest method of increasing muscle creatine stores appears to be to consume ~0.3 grams/kg/day of creatine monohydrate for at least 3 days followed by 3–5 g/d thereafter to maintain elevated stores. Ingesting smaller amounts of creatine monohydrate (e.g., 2–3 g/d) will increase muscle creatine stores over a 3–4 week period, however, the performance effects of this method of supplementation are less supported.

8. Creatine products are readily available as a dietary supplement and are regulated by the *U.S. Food and Drug Administration (FDA)*. Specifically, in 1994, U.S. President Bill Clinton signed into law the Dietary Supplement Health and Education Act (DSHEA). DSHEA allows manufacturers/companies/brands to make structure-function claims; however, the law strictly prohibits disease claims for dietary supplements.

9. Creatine monohydrate has been reported to have a number of potentially beneficial uses in several clinical populations, and further research is warranted in these areas.

The following literature review has been prepared by the authors in support of the aforementioned position statement.

## Creatine Supplementation and Exercise: A Review of the Literature

### Introduction

The use of creatine as a sport supplement has been surrounded by both controversy and fallacy since it gained widespread popularity in the early 1990's. Anecdotal and media reports have often claimed that creatine usage is a dangerous and unnecessary practice; often linking creatine use to anabolic steroid abuse [1]. Many athletes and experts in the field have reported that creatine supplementation is not only beneficial for athletic performance and various medical conditions but is also clinically safe [2-5]. Although creatine has recently been accepted as a safe and useful ergogenic aid, several myths have been purported about creatine supplementation which include:

1. All weight gained during supplementation is due to water retention.

2. Creatine supplementation causes renal distress.

3. Creatine supplementation causes cramping, dehydration, and/or altered electrolyte status.

4. Long-term effects of creatine supplementation are completely unknown.

5. Newer creatine formulations are more beneficial than creatine monohydrate (CM) and cause fewer side effects.

6. It's unethical and/or illegal to use creatine supplements.

While these myths have been refuted through scientific investigation, the general public is still primarily exposed to the mass media which may or may not have accurate information. Due to this confounding information, combined with the fact that creatine has become one of the most popular nutritional supplements on the market, it is important to examine the primary literature on supplemental creatine ingestion in humans. The purpose of this review is to determine the present state of knowledge concerning creatine supplementation, so that reasonable guidelines may be established and unfounded fears diminished in regard to its use.

### Background

Creatine has become one of the most extensively studied and scientifically validated nutritional ergogenic aids for athletes. Additionally, creatine has been evaluated as a potential therapeutic agent in a variety of medical conditions such as Alzheimer's and Parkinson's diseases. Biochemically speaking, the energy supplied to rephosphorylate adenosine diphosphate (ADP) to adenosine triphosphate (ATP) during and following intense exercise is largely dependent on the amount of phosphocreatine (PCr) stored in the muscle [6,7]. As PCr stores become depleted during intense exercise, energy availability diminishes due to the inability to resynthesize ATP at the rate required to sustained high-intensity exercise [6,7]. Consequently, the ability to maintain maximal-effort exercise declines. The availability of PCr in the muscle may significantly influence the amount of energy generated during brief periods of high-intensity exercise. Furthermore, it has been hypothesized that increasing muscle creatine content, via creatine supplementation, may increase the availability of PCr allowing for an accelerated rate of resynthesis of ATP during and following high-intensity, short-duration exercise [6-12]. Theoretically, creatine supplementation during training may lead to greater training adaptations due to an enhanced quality and volume of work performed. In terms of potential medical applications, creatine is intimately involved in a number of metabolic pathways. For this reason, medical researchers have been investigating the potential therapeutic role of creatine supplementation in a variety of patient populations.

Creatine is chemically known as a non-protein nitrogen; a compound which contains nitrogen but is not a protein per se [13]. It is synthesized in the liver and pancreas from the amino acids arginine, glycine, and methionine [9,13,14]. Approximately 95% of the body's creatine is stored in skeletal muscle. Additionally, small amounts of creatine are also found in the brain and testes [8,15]. About two thirds of the creatine found in skeletal muscle is stored as phosphocreatine (PCr) while the remaining amount of creatine is stored as free creatine [8]. The total creatine pool (PCr + free creatine) in skeletal muscle averages about 120 grams for a 70 kg individual. However, the average human has the capacity to store up to 160 grams of creatine under certain conditions [7,9]. The body breaks down about 1 – 2% of the creatine pool per day (about 1–2 grams/day) into creatinine in the skeletal muscle [13]. The creatinine is then excreted in urine [13,16]. Creatine stores can be replenished by obtaining creatine in the diet or through endogenous synthesis of creatine from glycine, arginine, and methionine [17,18]. Dietary sources of creatine include meats and fish. Large amounts of fish and meat must be consumed in order to obtain gram quantities of creatine. Whereas dietary supplementation of creatine provides an inexpensive and efficient means of increasing dietary availability of creatine without excessive fat and/or protein intake.

### Supplementation Protocols and Effects on Muscle Creatine Stores

Various supplementation protocols have been suggested to be efficacious in increasing muscle stores of creatine. The amount of increase in muscle storage depends on the levels of creatine in the muscle prior to supplementation. Those who have lower muscle creatine stores, such as those who eat little meat or fish, are more likely to experience muscle storage increases of 20–40%, whereas those with relatively high muscle stores may only increase stores by 10–20% [19]. The magnitude of the increase in skeletal muscle creatine content is important because studies have reported performance changes to be correlated to this increase [20,21].

The supplementation protocol most often described in the literature is referred to as the "loading" protocol. This protocol is characterized by ingesting approximately 0.3 grams/kg/day of CM for 5 – 7 days (e.g., ≃5 grams taken four times per day) and 3–5 grams/day thereafter [18,22]. Research has shown a 10–40% increase in muscle creatine and PCr stores using this protocol [10,22]. Additional research has reported that the loading protocol may only need to be 2–3 days in length to be beneficial, particularly if the ingestion coincides with protein and/or carbohydrate [23,24]. Furthermore, supplementing with 0.25 grams/kg-fat free mass/day of CM may be an alternative dosage sufficient to increase muscle creatine stores [25].

Other suggested supplementation protocols utilized include those with no loading phase as well as "cycling" strategies. A few studies have reported protocols with no loading period to be sufficient for increasing muscle creatine (3 g/d for 28 days) [15] as well as muscle size and strength (6 g/d for 12 weeks) [26,27]. These protocols seems to be equally effective in increasing muscular stores of creatine, but the increase is more gradual and thus the ergogenic effect does not occur as quickly. Cycling protocols involve the consumption of "loading" doses for 3–5 days every 3 to 4 weeks [18,22]. These cycling protocols appear to be effective in increasing and maintaining muscle creatine content before a drop to baseline values, which occurs at about 4–6 weeks [28,29].

### Creatine Formulations and Combinations

Many forms of creatine exist in the marketplace, and these choices can be very confusing for the consumer. Some of these formulations and combinations include creatine phosphate, creatine + β-hydroxy-β-methlybutyrate (HMB), creatine + sodium bicarbonate, creatine magnesium-chelate, creatine + glycerol, creatine + glutamine, creatine + β-alanine, creatine ethyl ester, creatine with cinnulin extract, as well as "effervescent" and "serum formulations". Most of these forms of creatine have been reported to be no better than traditional CM in terms of increasing strength or performance [30-38]. Reliable studies are yet to be published for creatine ethyl ester and creatine with cinnulin extract. Recent studies do suggest, however, that adding β-alanine to CM may produce greater effects than CM alone. These investigations indicate that the combination may have greater effects on strength, lean mass, and body fat percentage; in addition to delaying neuromuscular fatigue [31,32].

Three alternative creatine formulations have shown promise, but at present do not have sufficient evidence to warrant recommendation in lieu of CM. For example, creatine phosphate has been reported to be as effective as CM at improving LBM and strength, [36] yet this has only been reported in one study. In addition, creatine phosphate is currently more difficult and expensive to produce than CM. Combining CM with sodium phosphate, which has been reported to enhance high-intensity endurance exercise, may be a more affordable alternative to creatine phosphate. Secondly, a creatine/HMB combination was reported to be more effective at improving LBM and strength than either supplement alone [39], but other data has reported the combination offers no benefit in terms of increasing aerobic or anaerobic capacity [40,41]. The conflicting data therefore do not warrant recommendation of the creatine/HMB combination in lieu of CM. Lastly, creatine + glycerol has been reported to increase total body water as a hyper-hydration method prior to exercise in the heat, but this is also the first study of its kind. In addition, this combination failed to improve thermal and cardiovascular responses to a greater extent than CM alone [42].

The addition of nutrients that increase insulin levels and/or improve insulin sensitivity has been a major source of interest in the last few years by scientists looking to optimize the ergogenic effects of creatine. The addition of certain macronutrients appears to significantly augment muscle retention of creatine. Green et al. [24] reported that adding 93 g of carbohydrate to 5 g of CM increased total muscle creatine by 60%. Likewise, Steenge et al. [23] reported that adding 47 g of carbohydrate and 50 g of protein to CM was as effective at promoting muscle retention of creatine as adding 96 g of carbohydrate. Additional investigations by Greenwood and colleagues [30,43] have reported increased creatine retention from the addition of dextrose or low levels of D-pinitol (a plant extract with insulin-like properties). While the addition of these nutrients has proved to increase muscle retention, several recent investigations have reported these combinations to be no more effective at improving muscle strength and endurance or athletic performance [44-46]. Other recent studies, however, have indicated a potential benefit on anaerobic power, muscle hypertrophy, and 1 RM muscle strength when combining protein with creatine [47,48]. It appears that combining CM with carbohydrate or carbohydrate and protein produces optimal results. Studies suggest that increasing skeletal muscle creatine uptake may enhance the benefits of training.

### Effects of Supplementation on Exercise Performance and Training Adaptations

CM appears to be the most effective nutritional supplement currently available in terms of improving lean body mass and anaerobic capacity. To date, several hundred peer-reviewed research studies have been conducted to evaluate the efficacy of CM supplementation in improving exercise performance. Nearly 70% of these studies have reported a significant improvement in exercise capacity, while the others have generally reported non-significant gains in performance [49]. No studies have reported an ergolytic effect on performance although some have suggested that weight gain associated with CM supplementation could be detrimental in sports such as running or swimming. The average gain in performance from these studies typically ranges between 10 to 15% depending on the variable of interest. For example, short-term CM supplementation has been reported to improve maximal power/strength (5–15%), work performed during sets of maximal effort muscle contractions (5–15%), single-effort sprint performance (1–5%), and work performed during repetitive sprint performance (5–15%) [49]. Long-term CM supplementation appears to enhance the overall quality of training, leading to 5 to 15% greater gains in strength and performance [49]. Nearly all studies indicate that "proper" CM supplementation increases body mass by about 1 to 2 kg in the first week of loading [19].

The vast expanse of literature confirming the effectiveness of CM supplementation is far beyond the scope of this review. Briefly, short-term adaptations reported from CM supplementation include increased cycling power, total work performed on the bench press and jump squat, as well as improved sport performance in sprinting, swimming, and soccer [38,50-57]. Long-term adaptations when combining CM supplementation with training include increased muscle creatine and PCr content, lean body mass, strength, sprint performance, power, rate of force development, and muscle diameter [39,54-60]. In long-term studies, subjects taking CM typically gain about twice as much body mass and/or fat free mass (i.e., an extra 2 to 4 pounds of muscle mass during 4 to 12 weeks of training) than subjects taking a placebo [61-64]. The gains in muscle mass appear to be a result of an improved ability to perform high-intensity exercise via increased PCr availability and enhanced ATP synthesis, thereby enabling an athlete to train harder and promote greater muscular hypertrophy via increased myosin heavy chain expression possibly due to an increase in myogenic regulatory factors myogenin and MRF-4 [26,27,65]. The tremendous numbers of investigations conducted with positive results from CM supplementation lead us to conclude that it is the most effective nutritional supplement available today for increasing high-intensity exercise capacity and building lean mass.

### Medical Safety of Creatine Supplementation

While the only clinically significant side effect reported in the research literature is that of weight gain [4,18,22], many anecdotal claims of side effects including dehydration, cramping, kidney and liver damage, musculoskeletal injury, gastrointestinal distress, and anterior (leg) compartment syndrome still exist in the media and popular literature. While athletes who are taking CM may experience these symptoms, the scientific literature suggests that these athletes have no greater, and a possibly lower, risk of these symptoms than those not supplementing with CM [2,4,66,67].

Many of these fears have been generated by the media and data taken from case studies (n = 1). Poortmans and Francaux reported that the claims of deleterious effects of creatine supplements on renal function began in 1998 [68]. These claims followed a report that creatine supplementation was detrimental to renal glomerular filtration rate (GFR) in a 25-year-old man who had previously presented with kidney disease (glomerulosclerosis and corticosteroid-responsive nephritic syndrome) [69]. Three days later, a French sports newspaper, *L'Equipe*, reported that supplemental creatine is dangerous for the kidneys in any condition [70]. Several European newspapers then picked up the "news" and reported the same. Since that time, other individual case studies have been published posing that CM supplementation caused deleterious effects on renal function [71,72].

Much of the concern about CM supplementation and renal function has centered around concerns over increased serum creatinine levels. While creatinine does make up a portion of GFR and must be excreted by the kidneys, there is no evidence to support the notion that normal creatine intakes (< 25 g/d) in healthy adults cause renal dysfunction. In fact, Poortmans et al. have shown no detrimental effects of short- (5 days), medium- (14 days), or long-term (10 months to 5 years) CM supplementation on renal function [5,73,74]. Interestingly, Kreider et al. [4] observed no significant difference in creatinine levels between CM users and controls, yet most athletes (regardless of whether taking CM or not) had elevated creatinine levels along with proper clearance during intense training. The authors noted that if serum creatinine was examined as the sole measure of renal function, it would appear that nearly all of the athletes (regardless of CM usage) were experiencing renal distress. Although case studies have reported problems, these large-scale, controlled studies have shown no evidence indicating that CM supplementation in healthy individuals is a detriment to kidney functioning.

Another anecdotal complaint about supplemental creatine is that the long-term effects are not known. Widespread use of CM began in the 1990's. Over the last few years a number of researchers have begun to release results of long-term safety trials. So far, no long-term side effects have been observed in athletes (up to 5 years), infants with creatine synthesis deficiency (up to 3 years), or in clinical patient populations (up to 5 years) [4,5,18,75,76]. One cohort of patients taking 1.5 – 3 grams/day of CM has been monitored since 1981 with no significant side effects [77,78]. In addition, research has demonstrated a number of potentially helpful clinical uses of CM in heart patients, infants and patients with creatine synthesis deficiency, patients suffering orthopedic injury, and patients with various neuromuscular diseases. Potential medical uses of supplemental creatine have been investigated since the mid 1970s. Initially, research focused on the role of CM and/or creatine phosphate in reducing heart arrhythmias and/or improving heart function during ischemic events [18]. Interest in medical uses of creatine supplements has expanded to include those with creatine deficiencies [79-81], brain and/or spinal cord injuries [82-86], muscular dystrophy [87-90], diabetes [91], high cholesterol/triglyceride levels [92], and pulmonary disease [93] among others. Although more research is needed to determine the extent of the clinical utility, some promising results have been reported in a number of studies suggesting that creatine supplements may have therapeutic benefit in certain patient populations. In conjunction with short- and long-term studies in healthy populations, this evidence suggests that creatine supplementation appears to be safe when taken within recommended usage guidelines.

### Creatine Use in Children and Adolescents

Opponents of creatine supplementation have claimed that it is not safe for children and adolescents [1]. While fewer investigations have been conducted in using younger participants, no study has shown CM to have adverse effects in children. In fact, long-term CM supplementation (e.g., 4 – 8 grams/day for up to 3 years) has been used as an adjunctive therapy for a number of creatine synthesis deficiencies and neuromuscular disorders in children. Clinical trials are also being conducted in children with Duschenne muscular dystrophy [87,88]. However, as less is known about the effects of supplemental creatine on children and adolescents, it is the view of the ISSN that younger athletes should consider a creatine supplement only if the following conditions are met [19]:

1. The athlete is past puberty and is involved in serious/competitive training that may benefit from creatine supplementation;

2. The athlete is eating a well-balanced, performance-enhancing diet;

3. The athlete and his/her parents understand the truth concerning the effects of creatine supplementation;

4. The athlete's parents approve that their child takes supplemental creatine;

5. Creatine supplementation can be supervised by the athletes parents, trainers, coaches, and/or physician;

6. Quality supplements are used; and,

7. The athlete does not exceed recommended dosages.

If these conditions are met, then it would seem reasonable that high school athletes should be able to take a creatine supplement. Doing so may actually provide a safe nutritional alternative to illegal anabolic steroids or other potentially harmful drugs. Conversely, if the above conditions are not met, then creatine supplementation may not be appropriate. It appears that this is no different than teaching young athletes' proper training and dietary strategies to optimize performance. Creatine is not a panacea or short cut to athletic success. It can, however, offer some benefits to optimize training of athletes involved in intense exercise in a similar manner that ingesting a high-carbohydrate diet, sports drinks, and/or carbohydrate loading can optimize performance of an endurance athlete.

### The Ethics of Creatine

Several athletic governing bodies and special interest groups have questioned whether it is ethical for athletes to take creatine supplements as a method of enhancing performance. Since research indicates that CM can improve performance, and it would be difficult to ingest enough creatine from food in the diet, they rationalize that it is unethical to do so. In this age of steroid suspicion in sports, some argue that if you allow athletes to take creatine, they may be more predisposed to try other dangerous supplements and/or drugs. Still others have attempted to directly lump creatine in with anabolic steroids and/or banned stimulants and have called for a ban on the use of CM and other supplements among athletes. Finally, fresh off of the ban of dietary supplements containing ephedra, some have called for a ban on the sale of CM citing safety concerns. Creatine supplementation is not currently banned by any athletic organization although the NCAA does not allow institutions to provide CM or other "muscle building" supplements to their athletes (e.g., protein, amino acids, HMB, etc). In this case, athletes must purchase creatine containing supplements on their own. The International Olympic Committee considered these arguments and ruled that there was no need to ban creatine supplements since creatine is readily found in meat and fish and there is no valid test to determine whether athletes are taking it. In light of the research that has been conducted with CM, it appears that those who call for a ban on it are merely familiar with the anecdotal myths surrounding the supplement, and not the actual facts. We see no difference between creatine supplementation and ethical methods of gaining athletic advantage such as using advanced training techniques and proper nutritional methods. Carbohydrate loading is a nutritional technique used to enhance performance by enhancing glycogen stores. We see no difference between such a practice and supplementing with creatine to enhance skeletal muscle creatine and PCr stores. If anything, it could be argued that banning the use of creatine would be unethical as it has been reported to decrease the incidence of musculoskeletal injuries [2,66,75,94], heat stress [2,95,96], provide neuroprotective effects [82,83,85,97,98], and expedite rehabilitation from injury [86,99,100].

## Conclusion

It is the position of the International Society of Sports Nutrition that the use of creatine as a nutritional supplement within established guidelines is safe, effective, and ethical. Despite lingering myths concerning creatine supplementation in conjunction with exercise, CM remains one of the most extensively studied, as well as effective, nutritional aids available to athletes. Hundreds of studies have shown the effectiveness of CM supplementation in improving anaerobic capacity, strength, and lean body mass in conjunction with training. In addition, CM has repeatedly been reported to be safe, as well as possibly beneficial in preventing injury. Finally, the future of creatine research looks bright in regard to the areas of transport mechanisms, improved muscle retention, as well as treatment of numerous clinical maladies via supplementation.
